# CoCO_3_ from one-step micro-emulsion method as electrode materials for Faradaic capacitors

**DOI:** 10.1038/s41598-017-02004-8

**Published:** 2017-05-17

**Authors:** Yanfang Wang, Zheng Chang, Yi Zhang, Bingwei Chen, Lijun Fu, Yusong Zhu, Lixin Zhang, Yuping Wu

**Affiliations:** 1 School of Energy Science and Engineering & Institute for Electrochemical Energy Storage, Nanjing Tech University, Nanjing, 211816 China; 2 New Energy and Materials Laboratory (NEML), Department of Chemistry and Shanghai Key Laboratory of Molecular Catalysis and Innovative Materials, Fudan University, Shanghai, 200433 China

## Abstract

Faradaic capacitor (FC) has been widely investigated during the past few decades, and dozens of prototypes have been proposed. However, it has not reached its full potential. In this work, we demonstrate a kind of FC comprising of a CoCO_3_ electrode. Synthesized through a micro-emulsion route, such CoCO_3_ shows low crystallinity and porous wool-ball structures stacked by needle-like individuals. It shows desirable electrochemical properties in terms of excellent high-rate performance and high reversibility. Also, it could deliver a capacitance of 440 F·g^−1^ at 1 A·g^−1^, and shows no capacitance decay after 1000 cycles. Since metal carbonate is capable of delivering good electrochemical performances and its preparation is easier and more cost-efficient, it should be a feasible candidate for electrode material of FC.

## Introduction

With the highly increasing demands for energy consumption, various alternative energy sources having attractive features have come into researchers’ sights. Among them, secondary energy resources such as wind and solar energy are thought to be the most appealing candidates thanks to their renewability and cleanliness. However, harnessing energy from such sources is not always conceivable because various factors like location and climate could influence this process^1^. Scientists having different backgrounds and specialties, in order to better the utilization of these changeable energy resources, have demonstrated storing such intermittent energy in electrical energy storage (EES) devices to be a feasible solution. During the past decades, dozens of EES devices such as M-ion batteries (M = Li, Na and Al)^2–5^, metal-air batteries^6–8^, metal-sulfur batteries^9–12^, redox flow batteries and supercapacitors^13–15^ have been reported.

Electrochemical capacitor (EC), also called as supercapacitor, has drawn special attentions thanks to its fascinating properties, e.g. fast response, durable cycling ability and high power density^16^. Typically, there are two types of ECs which are classified according to their working mechanisms, i.e. electrochemical double-layer capacitor (EDLC) and Faradaic capacitor (FC, or battery-type capacitor)^17^. For EDLCs, the electrode materials usually are porous carbons having no electrochemical activity. In other words, only physical charge accumulation occurs at the electrode/electrolyte interface during the charge and discharge processes. For Faradaic capacitor (FC) or pseudocapacitor, charge storage and release are always accompanied by fast and reversible reactions happening on the surface or in near-surface areas. In this case, the electrode materials are electrochemically active. Generally, due to their different mechanisms, the EDLCs display higher rate capability and better cycling stability, but lower specific capacitance and energy density than those of the FCs. Trying to praise one more than the other is unreasonable, and it is clear that both of them have not reached their full potentials.

Hitherto, various materials such as transition metal oxides, metal hydroxides and conductive polymers have been investigated as electrode materials for FCs^18^. Taking Co_3_O_4_ as an example, in order to fabricate effective structures, various ways such as template-method, hydrothermal method and electrochemical deposition were explored^19–21^. However, such methods usually involve at least two steps. One is the preparation for precursors such as Co(OH)_2_. Following is the conversion process from precursors to final products, in which calcination is needed and more energy is consumed. In fact, the extra energy could be saved by leaving out of the thermal treatment. Actually, during the past few years, calcination-free Co(OH)_2_ has attracted widely research interests since it could be prepared in more cost-effective routes and is able to deliver good performances^22–26^.

Herein, inspired by the success of Co(OH)_2_, we have tried to demonstrate the possibility of fabricating FCs by using CoCO_3_ electrodes, and it ends up to be a good attempt. In this work, a kind of “wool-ball” like CoCO_3_ possessing amorphous and porous structure has been synthesized by using a soft template method based on reverse micelles. When being tested as electrode for FC in aqueous alkaline solution (0.5 M KOH), it could deliver a reversible specific capacitance of more than 400 F·g^−1^ as well as a 100% capacitance retention after 1000 cycles (at the current density of 1 A·g^−1^). The reaction intermediates appearing in the charge-discharge processes have been well studied and the reaction mechanisms have been expounded and proved. The results of our study on CoCO_3_, to some extent, could be analogized to other metal carbonates such as MnCO_3_ and NiCO_3_.

## Results

**Figure 1:**
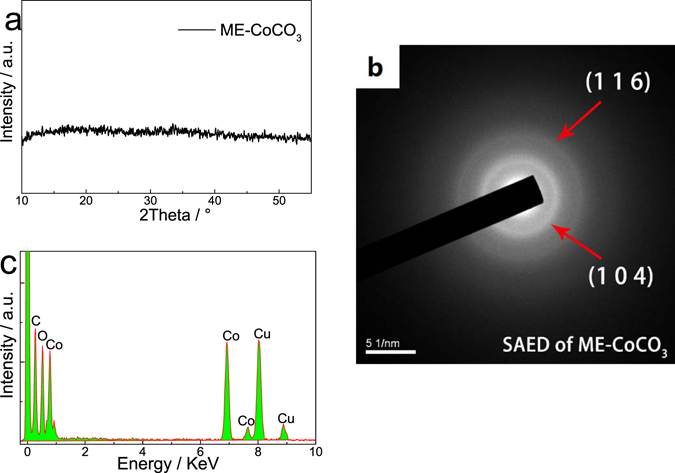
Some physical properties of the prepared ME-CoCO_3_: (**a**) XRD patterns (**b**) selected area electron diffraction (SAED) micrograph, and (**c**) energy dispersive X-ray spectroscopy (EDX) analyses.

Determined by the calcination-free routes, the X-ray diffraction patterns of the sample show dispersed diffraction peaks, demonstrating that it has low crystallinity (Fig. 1a). Two diffused halo rings observed in the selected area electron diffraction (SAED) patterns, which can be indexed to (104) and (116) planes of CoCO_3_ (JPCDS 11-0692), also verify its low crystallinity (Fig. 1b). Materials having low crystallinities always need lower energy and show higher rate performance when going through chemical reactions since phase transformation is always accompanied by crystal conversion, but such process is not necessary for amorphous materials. As shown in Fig. 1c, the atomic ratio of Co and O is calculated to be 1:2.71 based on the results of energy dispersive X-ray spectroscopy (EDX). Besides, the as prepared CoCO_3_ was tested by using a thermogravimetric-mass spectrum (TG-MS) combined system (Figure S1). As shown in the TG curve, there is 10% weight loss as the temperature increases to 175 °C. The signal of CO_2_ (m/z = 44) shows a peak in the region of 75–175 °C, which means that CoCO_3_ begins to decompose. Then, a sharp weight loss appears beginning at 200 °C, and it is accompanied by the release of large amounts of CO_2_ (the strong peak at around 275 °C in the mass spectra). The total weight loss is about 35%, which is consistent with the decomposition of CoCO_3_ into CoO. Thus, the sample is mainly consisted of CoCO_3_. If xCoCO_3_·yCo(OH)_2_·zH_2_O exists, its amount is very small.

**Figure 2:**
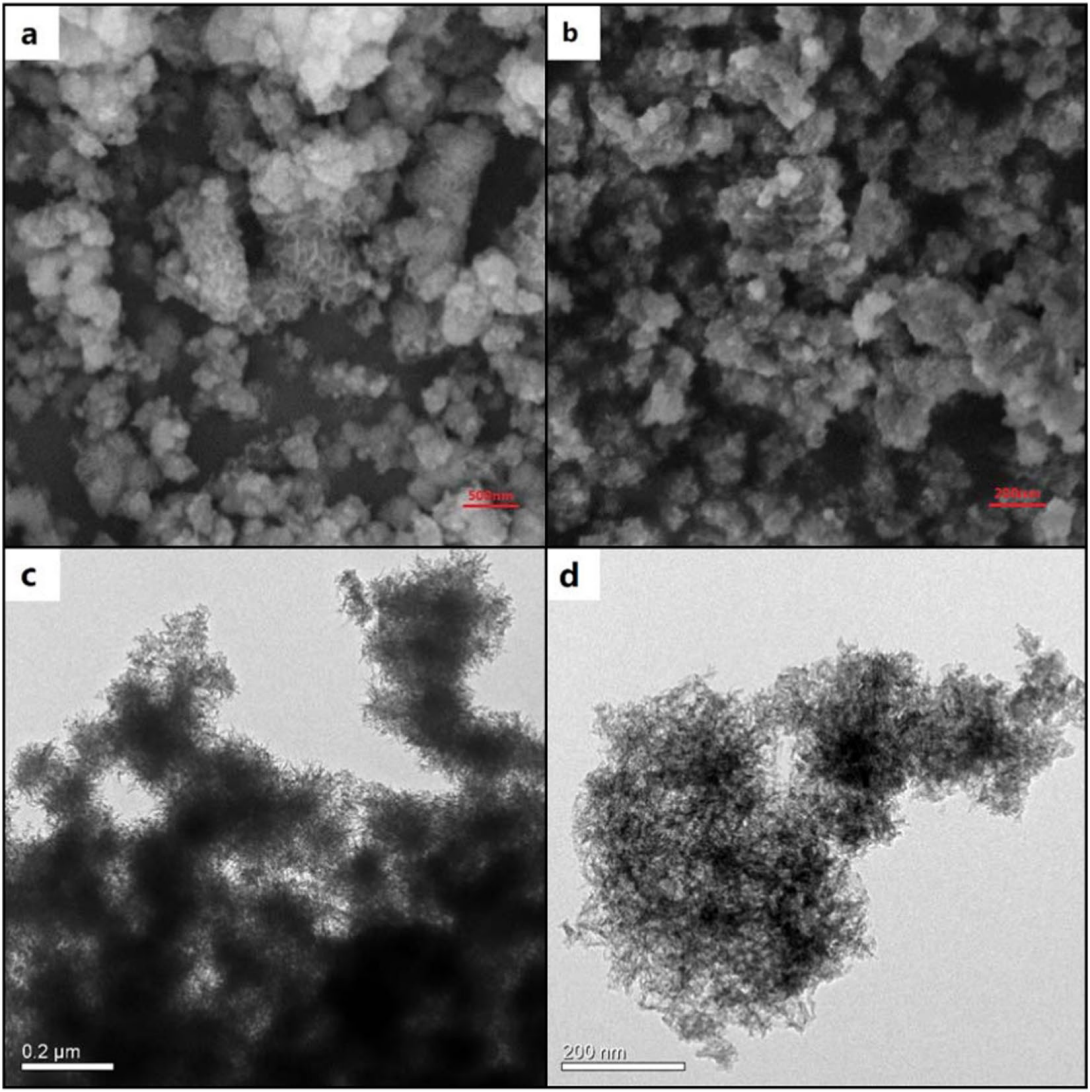
SEM micrographs of (**a**) ME-CoCO_3_ and (**b**) CP-CoCO_3_, (**c**) low and (**d**) high magnification TEM micrographs of ME-CoCO_3_.

Figure 2a and b show scanning electron microscopy (SEM) micrographs of CoCO_3_ samples synthesized through micro-emulsion (ME-CoCO_3_) and co-precipitation routes (CP-CoCO_3_), respectively. Both of them are nanoparticles with sizes of 100–500 nm. Specially, the ME-CoCO_3_ exhibits some uncommon surface structures which could be formed with the help of surfactants. As shown in the transmission electron microscopy (TEM) micrograph (Fig. 2c and Figure S2), the special surface structures could be determined to be needle-like CoCO_3_ individuals, which then accumulated to form “wool-ball” like nanoparticles. Since these individuals cannot stack compactly, CoCO_3_ nanoparticles tend to develop into porous structures, which could be confirmed by the enlarged TEM micrograph (Fig. 2d). Commonly, materials for FCs suffer from inevitable volumetric expansions during the charge and discharge processes, which then result in fast capacitance decline. Therefore, possessing porous structures means that the ME-CoCO_3_ could have enough space for expansion and thus could be able to have a long life span. Also, such porous structure could increase its specific surface areas which in turn could facilitate ion transfer and improve its rate performance. The nitrogen adsorption-desorption results reveal that ME-CoCO_3_ has a specific surface area (S_BET_) of 41.6 m^2^ g^−1^ which is much larger than that of CP-CoCO_3_ (23.1 m^2^ g^−1^). The higher specific surface area and porous structure allows it to possess larger interfaces connecting with conducting additives (acetylene black), which will lead to lower internal resistance.

**Figure 3:**
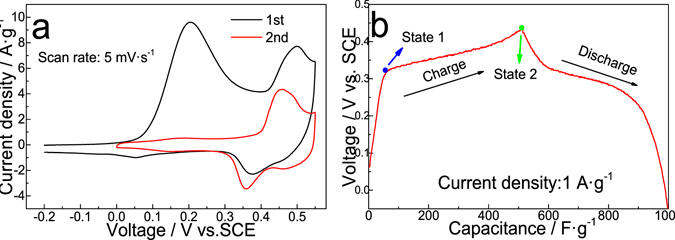
(**a**) CV curves and (**b**) charge-discharge curves of ME-CoCO_3_.

Figure 3a exhibits the cyclic voltammetry (CV) curves of ME-CoCO_3_. As shown, in the first cycle, two oxidation peaks centered at 0.2 and 0.48 V could be observed. Therefore, Co (II) should be oxidized to Co (IV) with an intermediate state of Co (III). In addition, the first peak has higher intensity in the first cycle, which means that large amounts of Co (II) have converted into Co (III) during this process. However, the reduction peak regarding the reaction from Co (III) to Co (II) shows much lower peak current density, demonstrating that such oxidation process (Co(II) to Co(III)) in the first cycle is irreversible. Clearly, in the second cycle, the intensity of first oxidation peak decreases sharply. Thus, the reversible reaction between Co(III) and Co(IV) dominates in the second and the following cycles.

**Figure 4:**
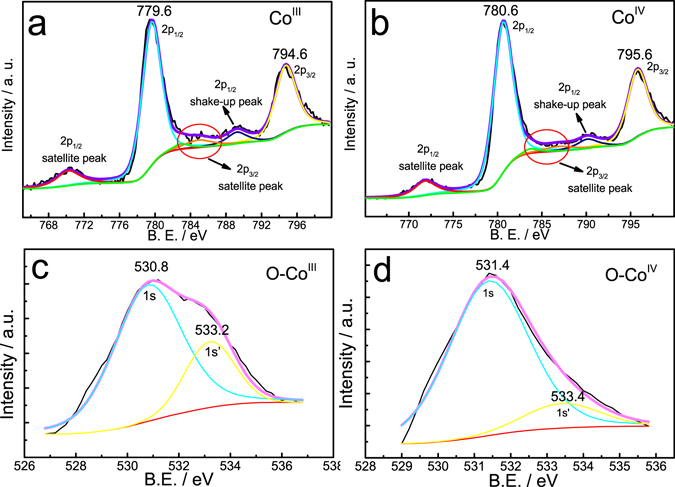
XPS spectra of (**a**) Co and (**c**) O at state 1 and those of (**b**) Co and (**d**) O at state 2 indicated in Fig. 3b.

Figure 3b shows the charge-discharge curves of ME-CoCO_3_ after CV test. The specific capacitance was calculated according to the following equation:

1 $${{\rm{C}}}_{m}={\rm{I}}\cdot t/({\rm{\Delta }}{\rm{V}}\cdot {\rm{m}})$$

where *C*_*m*_ is the specific capacitance of the electrode material (F·g^−1^), *I* is the charge/discharge current (A), *t* is the charge/discharge time (s), Δ*V* is the potential window, and *m* is the mass of electrochemical active material. A pair of plateaus could be observed in the charge-discharge curves, which are in accordance with the reversible conversion between Co(III) and Co (IV). Electrode materials were collected at the beginning of the plateau and at the end of the charge process, and were notated as state 1 and state 2, respectively. Then, such materials were characterized with XPS technique (Fig. 4). As shown in Fig. 4a, two typical peaks centered at 794.6 and 779.6 eV in the Co_2p_ spectra (Fig. 4a) are accordant with the Co_2p3/2_ and Co_2p1/2_ spin-orbit peaks of Co(III). In addition, there are some small peaks. The satellite peaks located at around 770 and 785 eV should be caused by some minor impurities, and the peak located at around 790 could be assigned to the shake-up peak of Co_2p1/2_. The split O_1s_ peaks centered at 530.8 and 533.2 eV indicate that O exists in two forms at state 1 (Fig. 4c). As for state 2, in the Co_2p_ spectra (Fig. 4b), two typical peaks centered at 795.6 and 780.6 eV could be assigned to the Co_2p3/2_ and Co_2p1/2_ spin-orbit peaks of Co(IV). In the O_1s_ spectra (Fig. 4d), the areas surrounded by the typical peak centered at 531.4 eV are much larger than that surrounded by the peak centered at 533.4 eV, implying that O exists in one major phase. Therefore, the main compound at state 1 and state 2 should be CoOOH and CoO_2_, respectively. Besides, the small peak of O_1s_ detected at 533.4 eV corresponds to the O in H_2_O.

According to the above results, the reaction mechanisms of CoCO_3_ in alkaline solutions can be proposed as follows:

2 $${{\rm{CoCO}}}_{3}+3{{\rm{OH}}}^{-}-{{\rm{e}}}^{-}\to {\rm{CoO}}({\rm{OH}})+{{\rm{H}}}_{2}{\rm{O}}+{{\rm{CO}}}_{3}^{2-}\,\,\,({\rm{irreversible}})$$

3 $${\rm{Co}}{({\rm{OH}})}_{2}+{{\rm{OH}}}^{-}-{{\rm{e}}}^{-}\leftrightarrow {\rm{CoO}}({\rm{OH}})+{{\rm{H}}}_{2}{\rm{O}}$$

4 $${\rm{CoO}}({\rm{OH}})+{{\rm{OH}}}^{-}-{{\rm{e}}}^{-}\leftrightarrow {{\rm{H}}}_{2}{\rm{O}}+{{\rm{CoO}}}_{2}$$

In alkaline solutions, cobalt carbonate would convert to cobalt hydroxide spontaneously according to the precipitation and dissolution equilibrium. Such process could be speeded up by forward current since the movement of ions could be promoted under current. Therefore, CoCO_3_ would convert to Co(OH)_2_ and CoOOH rapidly in the 1^st^ cycle (Equation 2), and such conversion is irreversible, which has been demonstrated by the CV curve. Then, during the following cycles, conversions between Co(OH)_2_/CoOOH and CoO_2_ would happen reversibly (equations 3 and 4).

**Figure 5:**
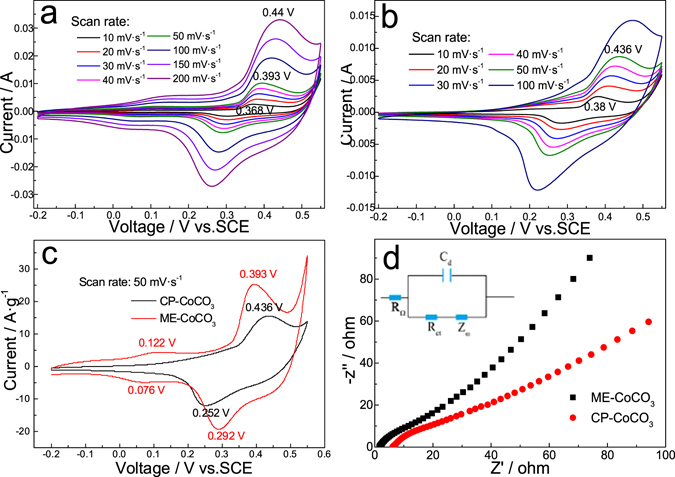
CV curves of (**a**) ME-CoCO_3_, (**b**) CP-CoCO_3_ at different scan rates and (**c**) both of them at the scan rate of 50 mV·s^−1^, and (**d**) their EIS results.

Figure 5a shows the CV curves of ME-CoCO_3_ at various potential scan rates. Two pairs of peaks could be seen at all scan rates, and the conversion between Co(III) and Co(IV) dominates in all cases. Specially, the CV curves maintain regular shapes even at a very high scan rate of 200 mV·s^−1^, showing that it has excellent high-rate performance. In the case of the CP-CoCO_3_, its CV curves show severe distortion with increasing scan rates (Fig. 5b). In addition, the symmetric CV curves prove that the redox reactions occurring in both electrodes are reversible.

The electrochemical impedance spectroscopy (EIS) results are shown in Fig. 5d. The internal resistance of an electrode (notated as *R*_*Ω*_ or *R*_*ESR*_), i.e. the point intersecting with real axis at high frequency, includes ionic resistance of electrolyte (*R*_*A*_), intrinsic resistance of electrode materials (*R*_*B*_) and contact resistance (*R*_*C*_, negligible due to the high conductivity of nickel mesh). The polarization resistance (*R*_*P*_), i.e. the diameter of semicircle, reveals penetrating ability of electrolyte into porous electrode. The diffusional resistance (*R*_*D*_), i.e. the length of 45° line in middle frequency region, indicates migration rate of ions from electrolyte inside pores to the electrode surface. The sum of *R*_*D*_ and *R*_*P*_ is notated as charge transfer resistance (*R*_*ct*_)^27^. Clearly, the ME-CoCO_3_ electrode presents smaller *R*_*ESR*_ (2.0 Ω) than the CP-CoCO_3_ (6.3 Ω), demonstrating its better electronic conductivity. Also, the *R*_ct_ of the ME-CoCO_3_ (15 Ω) is smaller than that of the CP-CoCO_3_ (28 Ω) thanks to its “wool-ball” like structure. Commonly, in narrow pores or nonporous structures, ion could not migrate smoothly become of large diffusion drag. In our ME-CoCO_3_ electrode, the porous structure piled up by needle-like individuals could provide free pathways for ions. In the low frequency range, the larger slope of the ME-CoCO_3_ electrode indicates its better capacitive behaviour.

**Figure 6:**
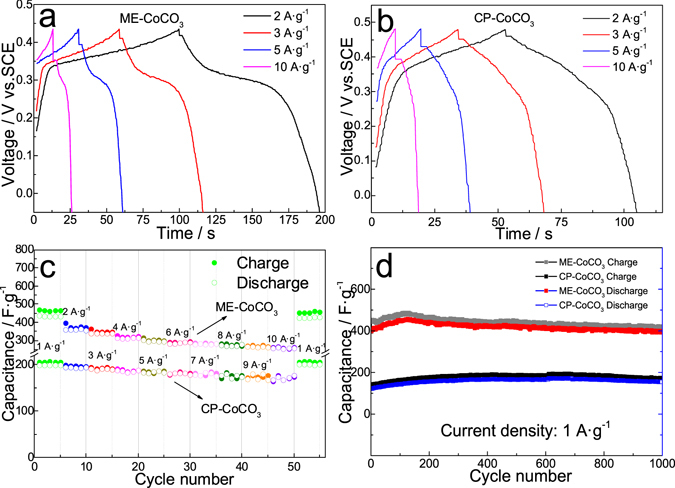
Galvanostatic charge-discharge curves of (**a**) ME-CoCO_3_ and (**b**) CP-CoCO_3_. (**c**) Rate performances and (**d**) cycling behaviours of ME-CoCO_3_ and CP-CoCO_3_.

Figure 6a and b show the galvanostatic charge-discharge curves of the ME-CoCO_3_ and CP-CoCO_3_ at various current densities. It can be seen that the charge and discharge times are nearly the same at a certain current density, indicating their good reversibility. Their rate and cycling performances were tested and shown in Fig. 6c. The ME-CoCO_3_ could deliver a capacitance of 440 F·g^−1^ at 1 A·g^−1^ and 260 F·g^−1^ at 10 A·g^−1^, which is even higher than that at 1 A·g^−1^ in the case of the CP-CoCO_3_ (155 F·g^−1^). As to the cycling behaviour (Fig. 6d), it can be seen that the specific capacitances of both CoCO_3_ gradually increase to peak values and then decrease slightly. Such increase at the initial cycling period is consistent with the activation process reported before^28^. At the beginning of the charge/discharge processes, the electrochemical active materials could not be fully wetted by the electrolyte and only the surface areas could be utilized for charge storage, and thus the transfers of ions and electrons could not be fully realized for the electrode. Along with the continuing intercalation and de-intercalation, active sites even in the near-surface bodies could be exploited. Therefore, the capacitance could be increased and thus the electrode could reach a so-called activated state. The activation process varies with the electrode material changes. In the case of the CP-CoCO_3_, the activation process lasted for 600 cycles. However, as for the ME-CoCO_3_, such process only took 150 cycles thanks to its porous structure and lower *R*_ct_. Then, the capacitance would decrease after reaching the ceiling value since extra stress and strain caused by volumetric expansion is inevitable in materials for FCs. Such extra stress and strain could result in structure collapse and thus reduce available active sites for charge storage. However, because of its enough space for volumetric expansion, such extra forces could not affect the ME-CoCO_3_ severely. So, it shows almost no capacitance decline after 1000 cycles at 1 A·g^−1^, which is desirable for EES applications. Figure S3 shows the long-term cycling performance of ME-CoCO_3_ after activation. It could deliver 95.3% of the initial specific capacitance after 4000 cycles at 5 A·g^−1^.

## Discussion

In this report, we propose the opinion of replacing metal oxides with metal carbonates. As we demonstrate in our paper, metal carbonates could be able to perform good properties as electrodes or pre-electrodes for supercapacitors, and they could be synthesized under mild conditions such as low temperature and pressure. Here, we demonstrate a kind of FC by using CoCO_3_ as electrode material and study the reaction mechanisms of CoCO_3_ carefully. The synthesis of such material includes a calcination-free route, so that such energy could be saved compared with the synthesis of Co_3_O_4_. Besides, with the help of surfactant, CoCO_3_ synthesized through micro-emulsion approach shows effective “wool-ball” like structures. Such porous structures could not only facilitate charge transfer but also provide enough space to avoid structure collapse caused by volumetric expansion. The reaction mechanisms could be explained by an irreversible conversion between Co(II) and Co(III) in the 1^st^ cycle followed by reversible reactions. Although the reactions in the following cycles might be similar to those for Co(OH)_2_ electrode, CoCO_3_ is also worthy of investigation because of the possibility it offers. Not to mention that CoCO_3_ would be able to meet the demands for EES devices. In the case of the ME-CoCO_3_, it presents excellent high-rate performance and high reversibility. Also, it delivers a capacitance up to 440 F·g^−1^ at 1 A·g^−1^, and shows no capacitance decay after 1000 cycles. Therefore, CoCO_3_ could be feasible candidates for FCs, and other metal carbonates such as MnCO_3_ and NiCO_3_ could also be available alternatives for EES applications.

## Methods

### Sample preparation

The CoCO_3_ nanoparticles were prepared through co-precipitation routes and micro-emulsion routes^29, 30^, and they are denoted as CP-CoCO_3_ and ME-CoCO_3_, respectively. In a typical process, a micro-emulsion system was first prepared by dissolving cetyltrimethyl ammonium bromide (CTAB, 4.0 g) in a mixture of cyclohexane (100 ml), *n*-pentanol (5.0 ml) and 0.1 M (NH_4_)HCO_3_ (7.5 ml) aqueous solutions. After stirring for 30 minutes, CTAB was dissolved in the solution absolutely. Then, 2.5 ml of 0.1 M Co(NO_3_)_2_ solution was added dropwise under stirring and a transparent micro-emulsion was obtained. Finally, the amaranth product was filtered, washed several times with ethanol and distilled water, and dried under 80 °C in a vacuum oven. The yield of the ME-CoCO_3_ in a typical process was about 30 mg, about 80%. The CP-CoCO_3_ was prepared by a co-precipitation method. An aqueous solution (25 ml of 0.1 M Co(NO_3_)_2_) was added dropwise into 30 ml 0.1 M Na_2_CO_3_ aqueous solutions under continuous stirring. The precipitate was collected, washed and dried under 80°C in a vacuum oven as well.

### Material characterization

X-ray diffraction (XRD) patterns were collected by using a BrukerD4 X-ray diffractometer (Bruker, Germany) with Ni-filtered Cu Kα radiation (40 kV, 40 mA). Field emission scanning electron microscopy (FE-SEM) was performed on a FE-SEM-4800-1. Prior to the FESEM analyses, a thin layer of Au was sputtered on the surfaces of the as-prepared materials. Transmission electron microscopy (TEM) was performed by using a JEOL JEM-2010 transmission electron microscope (JEOL, Japan) operated at 200 kV. X-ray photoelectron spectroscopy (XPS) was performed by using a PHI5300 X-ray photoelectron spectroscope (Perki-Elmer, America) with aluminum target (14 kV, 250 W). Thermogravimetric-mass spectrum test was performed on SDT Q600 (USA) – GSD 301 T2 (Germany) combined system under nitrogen flow.

### Electrochemical test

The as-prepared ME-CoCO_3_ or CP-CoCO_3_ was mixed with poly(tetrafluoroethylene) (PTFE) and acetylene black in a weight ratio of 8:1:1. The mixture was well-distributed in the ethanol with the help of ultrasonic treatment. After drying, the mixture was pressed into a film and cut into pieces. One piece with a certain mass was weighted and pressed onto a piece of nickel mesh with a mass loading of 10 mg cm^−2^ to act as the working electrode. A three-electrode system consisting of the above working electrode, nickel mesh as the counter electrode, and saturated calomel electrode (SCE) as the reference electrode was used to test the electrochemical behaviours of the electrode materials. The cyclic voltammetry (CV) measurements were tested on an electrochemical working station (CHI 440B). The charge/discharge behaviours including the rate and cycling performance were tested on a cell tester (Land, Wuhan, China).

## Electronic supplementary material

Supplementary Information
