# Supplementary material for: CoCO3 from one-step micro-emulsion method as electrode materials for Faradaic capacitors
Source: Sci Rep. 2017 May 17;7:2026. doi: 10.1038/s41598-017-02004-8 (PMC5435784; doi:10.1038/s41598-017-02004-8)
Supplement: Supplementary file 1 — Supplementary Information [file 41598_2017_2004_MOESM1_ESM.pdf]

Supplementary Information

$CoCO_3$ from one-step micro-emulsion method as electrode materials for Faradaic capacitors

Yanfang Wang[1,2], Zheng Chang[2], Yi Zhang[1], Bingwei Chen[2], Lijun Fu[1,*], Yusong Zhu[1,*], Lixing Zhang[2,*], and Yuping Wu[1,2,*]

[1] College of Energy, Nanjing Tech University, Nanjing 211816, China

[2] New Energy and Materials Laboratory (NEML), Department of Chemistry and Shanghai Key Laboratory of Molecular Catalysis and Innovative Materials, Fudan University, Shanghai 200433, China

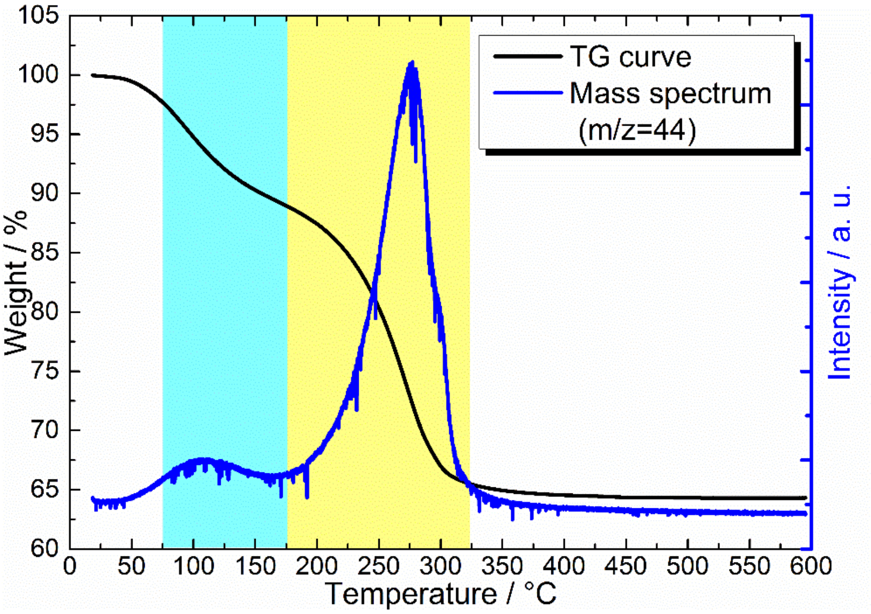

Figure S1. Thermogravimetric- mass spectrum results of ME-$CoCO_3$.

* Email: wuyp@njtech.edu.cn; wuyp@fudan.edu.cn

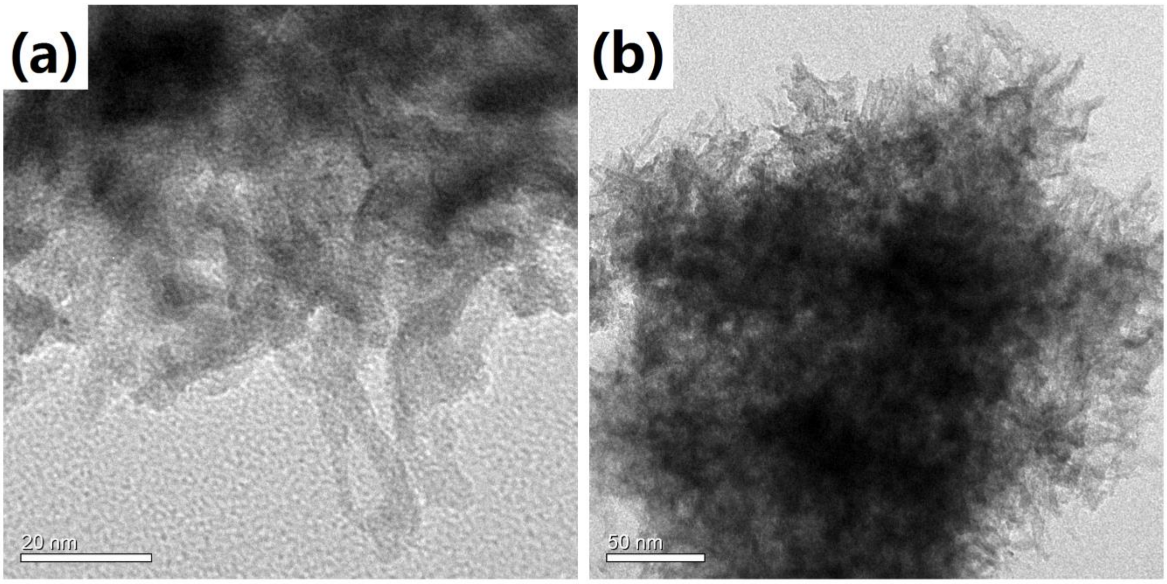

Figure S2. High-resolution TEM images of ME-$CoCO_3$.

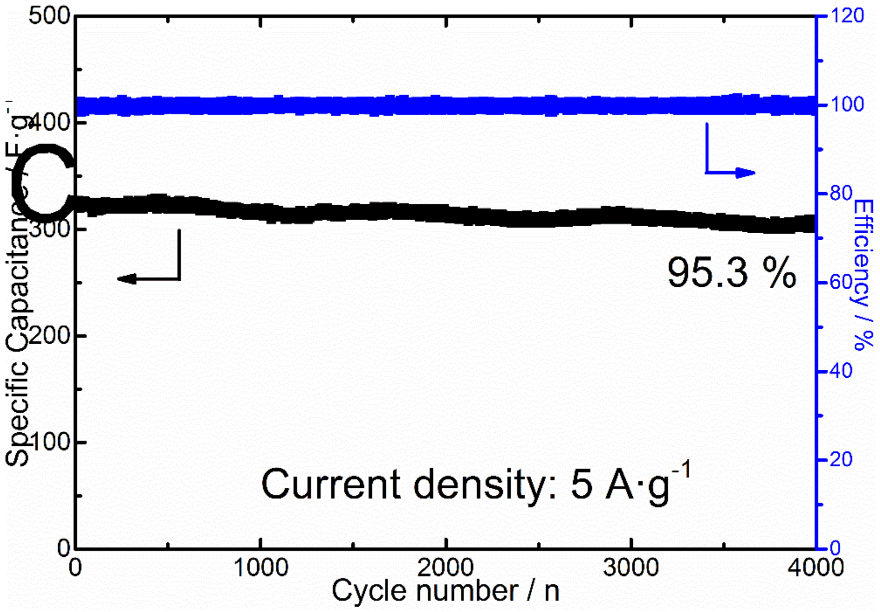

Figure S3. Cycling performance of ME-$CoCO_3$ after activation.
